# Comparison of practicability and effectiveness between unassisted HIV self-testing and directly assisted HIV self-testing in the Democratic Republic of the Congo: a randomized feasibility trial

**DOI:** 10.1186/s12879-020-05554-x

**Published:** 2020-11-11

**Authors:** Serge Tonen-Wolyec, Charles Kayembe Tshilumba, Salomon Batina-Agasa, Roland Marini Djang’eing’a, Marie-Pierre Hayette, Laurent Belec

**Affiliations:** 1 Ecole Doctorale Régionale D’Afrique Centrale en Infectiologie Tropicale, Franceville, Gabon; 2 Department of Internal Medicine, Faculty de Medicine, University of Bunia, Bunia, Democratic Republic of the Congo; 3 Department of Internal Medicine, Faculty of Medicine and Pharmacy, University of Kisangani, Kisangani, Democratic Republic of the Congo; 4 Department of Pharmaceutical Sciences, Laboratory of Analytical Chemistry, Faculty of Medicine, University of Liège, Liège, Belgium; 5 Department of Clinical Microbiology, University Hospital of Liège, Liege, Belgium; 6 Laboratory of virology, Hôpital Européen Georges Pompidou, and University of Paris Descartes, Paris Sorbonne Cité, Paris, France

**Keywords:** HIV, HIV self-testing, Unassisted HIV self-testing, Directly assisted HIV self-testing, Democratic Republic of the Congo

## Abstract

**Background:**

HIV self-testing (HIVST) can be performed using directly assisted and unassisted approaches in facilities or communities to reach different populations. The aim of this study was to compare the practicability and effectiveness of the two delivery approaches for HIVST, unassisted HIVST (UH) and directly assisted HIVST (DAH), in the field setting of Kisangani, the Democratic Republic of the Congo (DRC).

**Methods:**

A randomized (1:1), non-blinded, non-inferiority trial using a blood-based and facility-based HIVST method was carried out in four facilities in Kisangani, the DRC, targeting populations at high risk for HIV infection. The primary outcome was the difference in the practicability of the HIV self-test between the two arms. Practicability was defined as successfully performing the test and correctly interpreting the result. Requests for assistance, positivity rate, linkage to care, and willingness to buy an HIV self-test kit constituted the secondary outcomes for HIVST effectiveness. The adjusted risk ratios (aRRs) were calculated using Poisson regression.

**Results:**

The rate of successfully performing the test was same (93.2%) in the UH and DAH arms. The rate of correctly interpreting the results was 86.9% in the UH arm versus 93.2% in the DAH arm, for a difference of − 6.3%. After the follow-up 72 h later, participants in the UH arm had a significantly lower chance of correctly interpreting the test results than those in the DAH arm (aRR: 0.60; *P* = 0.019). Although the positivity rate was 3.4% among the participants in the DAH arm and 1.7% among those in the UH arm, no significant differences were found between the two arms in the positivity rate, requests for assistance, and linkage to care. Willingness to buy an HIV self-test was higher in the UH arm than in the DAH arm (92.3% versus 74.1%; aRR: 4.20; *P* < 0.001).

**Conclusion:**

The results of this study indicate that UH is as practicable and effective as DAH among individuals at high risk for HIV infection in Kisangani, the DRC. However, additional support tools need to be assessed to improve the interpretation of the self-test results when using the UH approach.

**Trial registration:**

PACTR201904546865585. Registered 03 April 2019 - Retrospectively registered, https://pactr.samrc.ac.za/TrialDisplay.aspx?TrialID=6032

## Background

Despite the progress in scaling up HIV testing worldwide, 25% of all people living with HIV remain unaware of their HIV status [1]. Based on recent modelling, it will be difficult to achieve the ambitious UNAIDS ‘90–90-90’ targets by 2020 unless efforts are increased and better targeted and innovations are used strategically [2, 3]. HIV self-testing (HIVST) is a novel innovation that can potentially increase the uptake of HIV testing and help control the HIV epidemic by 2030 by serving populations who live far from existing HIV testing services [4, 5].

HIVST can be performed through directly assisted and unassisted approaches in facilities or communities to reach different populations. According to the World Health Organization (WHO) [6], directly assisted HIVST (DAH) is when an individual self-tests for HIV and receives a face-to-face demonstration by a trained provider or peer of how to perform the test and interpret the result. This approach is recommended in cases where people with disabilities and low literacy skills require assistance. Unassisted HIVST (UH) is when an individual self-tests for HIV and performs the self-test guided only by the instructions for use provided by the manufacturer without assistance from a trained provider [6].

Whether HIV self-test kits can be used properly and the self-test results be interpreted correctly remain under debate [7–14]. Indeed, several studies in sub-Saharan Africa have assessed the ability of individuals to perform the HIV self-test and interpret the results according to different approaches as DAH [4, 15], UH [8, 10, 16, 17], and both together [18], identifying difficulties in collecting and transferring the sample and errors in interpreting the self-test results as the main barriers to successfully performing self-tests [9]. While errors in interpreting the self-test can be controlled in the DAH approach, they are difficult to control in the UH approach because of the lack of sufficient support tools [8, 9]. Unfortunately, misinterpreted self-tests could increase the risk of spreading HIV, especially when a positive result is read as negative [19]. Because few studies in the literature have compared the use of these two approaches to the distribution of HIV self-testing in the field, such comparisons are needed to clarify this issue, which will help to improve the implementation of HIV/STD prevention programs.

In the Democratic Republic of the Congo (DRC), 46% of those living with HIV do not know their HIV status [20], and the policy to support HIVST is under development [21]. Although some field evidence regarding the practicability and performance of DAH has been reported for the general population [8] and key populations such as female sex workers [13] and young adolescents [22], to our knowledge, no study has compared UH to DAH in the DRC. Thus, this study aimed to compare the practicability and effectiveness of HIVST using the UH and DAH approaches. A blood-based and facility-based HIVST method was used in a randomized, non-blinded, non-inferiority trial among a high-risk population for HIV infection acquisition in Kisangani, the DRC.

## Methods

### Study design and participants

This randomized implementation trial was conducted between August and November 2018 in Kisangani, the DRC. Kisangani, the capital city of Tshopo Province, is the third-largest urbanized city in the DRC, with 1.6 million inhabitants and a 2.3% HIV seroprevalence in the general public aged 15 to 49 years [22].

Trained research assistants (physicians or nurses) enrolled participants at four facilities (University Hospital of Kisangani, General Hospital of Kabondo, and the health centres of Neema and Saint Joseph). These facilities were selected because they integrate HIV prevention and care packages, provide free care to people living with HIV, and provide convenient access for those at high risk of exposure in the transmission hotspots in Kisangani. The survey was promoted and made visible by placing posters in the facilities and by informing people in the transmission hotspots in Kisangani and distributing tokens redeemable at the clinics to anyone both during the day and at night.

Participants were eligible for the study if they were between 18 and 49 years old, were at high risk of acquiring HIV infection, did not know their HIV status, lived or worked in Kisangani for at least 6 months before enrolment, and were available and accessible by phone. High risk of acquiring HIV infection was defined as being sexually active with a history of unprotected intercourse with one or more partners of unknown HIV serostatus within the past 6 months, having had new sex partners in the past 6 months, having symptoms of sexually transmitted infections (STIs) in the same period, engaging in commercial sex activities, or being in a known HIV discordant partnership [18].

### Randomization procedures

**Fig. 1:**
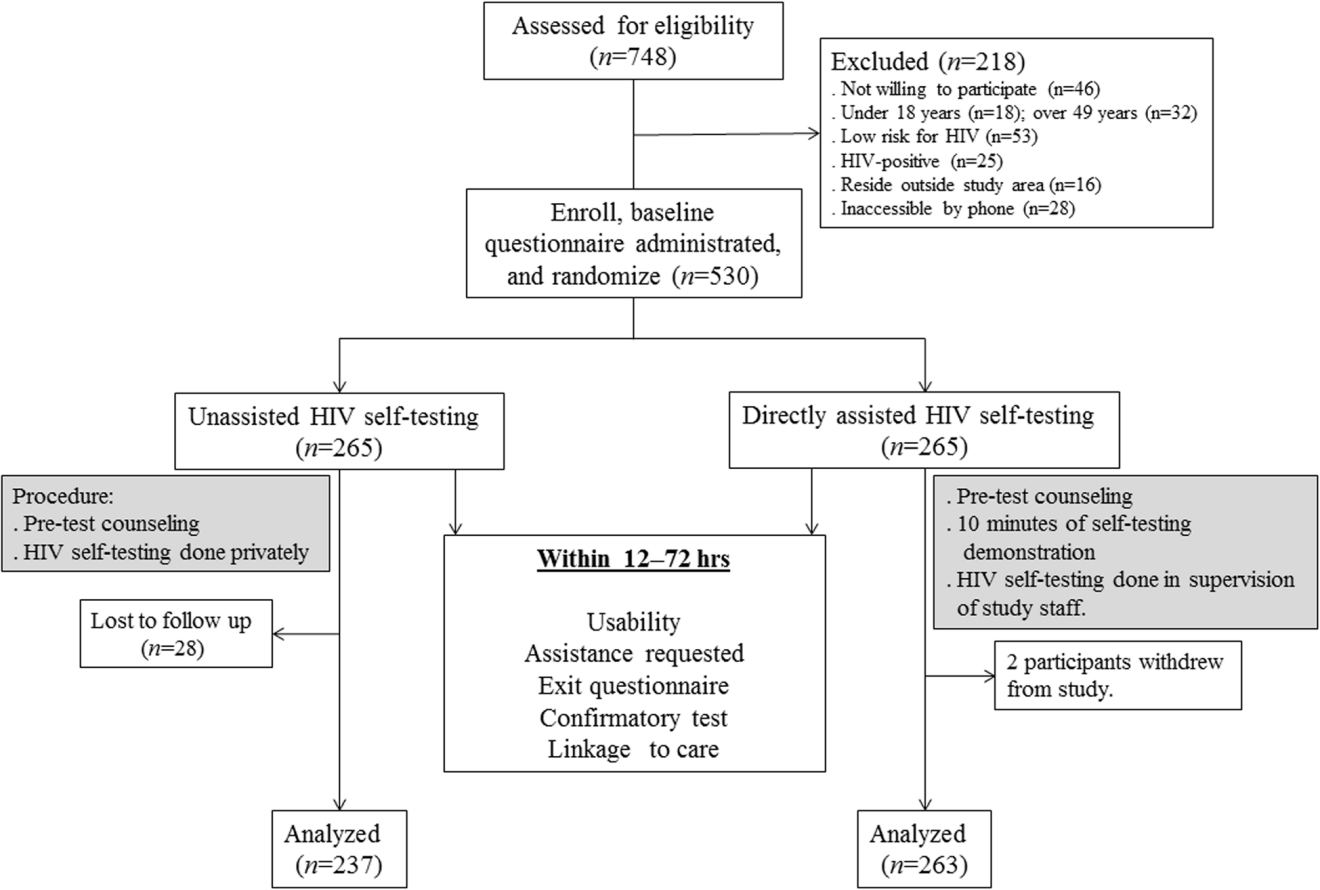
Flow charts showing enrolment, randomization and follow-up of study participants

Participants were randomized at a ratio of 1:1 through block randomization (block size 4, 6, 8). Eligible participants were randomly assigned to one of two self-testing groups (Fig. 1), DAH or UH, using sequentially numbered sealed randomization envelopes. Because of the nature of the intervention, the study participants and study staff could not be blinded. However, the study staff and participants were unaware of the assignment until the envelope was opened.

### Study procedures and data collection

The blood-based HIVST was performed using the Exacto® Test HIV (Biosynex, Strasbourg, France) self-test kit, which includes simplified pictorial instructions printed in colour in A3 format for use in French, Lingala, and Swahili, as previously reported [8]. After obtaining written informed consent and before randomization, the participants completed a self-administered baseline questionnaire to collect data on their demographic characteristics, sexual behaviour, and HIV testing history, after which they received adequate pre-test HIV counselling.

In the DAH arm, a brief, the participants watched a 10 min face-to-face demonstration of how to use the self-test to familiarize them with the contents of the self-test kit. After this, the participant was asked to perform the HIV self-test in a private room supervised by trained research assistants (supervisors). After completing the test, the participant completed a practicability report using a standardized sheet. The supervisor-interpreted results and the requests for verbal assistance were recorded by the supervisor on a standardized sheet. The supervisors had received rigorous training on how to talk to the participants when they asked for verbal assistance when performing the HIV self-test.

In the UH arm, the participants were asked to perform the HIV self-test at home or in a convenient private location and then read the results guided only by the test’s instructions without the 10 min demonstration or supervision. The participants were instructed to complete the practicability report using the standardized sheet within 10 min of performing the self-test. Furthermore, the participants were invited to return with the test cassette and the standardized sheet (placed in a sealed envelope) to the facility within 12–72 h to re-read the test results and undergo an additional evaluation. Telephone assistance was offered to the participants if needed. The need for assistance from a trusted person was self-reported by the participants and recorded by the investigators.

In each study arm, a confirmatory HIV test using a national rapid test algorithm [21, 22] was performed after HIVST if the test was reactive. If seropositivity was confirmed, the participants were referred to care services. Post-test counselling was provided to the participants if needed. The standardized sheet included questions that asked the participant to confirm the presence of blood in the square well of the test, describe the appearance of the control strip in the self-test, and report the final interpreted self-test result. The final results were recorded as one of three outcomes: (i) may have HIV (preliminary positive), (ii) do not have HIV (preliminary negative), and (iii) test not working (invalid). There was a 24 h helpline for the participants in which anonymity was assured by instructing the participants to introduce themselves using their three-character randomization code. The investigator recorded all information about the provided telephone assistance on a follow-up sheet.

An exit questionnaire was self-administered after completing all parts of the testing process. The questionnaire assessed satisfaction, the willingness to purchase an HIV self-test kit, and the unit purchase price of the test in United States dollars (USD).

### Study outcomes

The primary outcome was the difference in the practicability of the Exacto® Test HIV (Biosynex) self-test kit between the UH and DAH approaches. Practicability was defined as the successful performance of the HIV self-test and the correct interpretation of the HIV self-test result. The successful performance of the HIV self-test was determined by the presence of the control strip. The self-test result was interpreted as negative when the Control line (C) was present and readable and the Test line (T) was absent. The result was positive when the ‘C’ and ‘T’ (clearly or poorly readable) lines were present, and it was invalid when the ‘C’ line was absent regardless of the presence or absence of the ‘T’ line.

Secondary outcomes included the proportion of participants who requested assistance, the retention rate, positivity rate with confirmation HIV testing, linkage to care, and willingness to buy the HIV self-test kit if locally available. The retention rate was defined as the number of included participants who completed the entire evaluation process through the follow-up period. We used the above secondary outcomes as our measures of the effectiveness of HIVST between the UH to the DAH approaches under field conditions in the DRC.

### Sample size

A one-sided design to test non-inferiority between the groups was used; specifically, the hypothesis that the practicability of UH is objectively non-inferior to that of DAH was tested. The sample size was estimated using the following formula: *n* = 1/Δ_L_^2^ (Z_1-α_ √[π_Ν_ (1-π_Ν_) + π_R_ (1–π_R_)] + Z_1-β_ √[2π_R_ (1 – π_R_)])^2^, where πN and πR are the success proportions for UH and DAH, respectively, with α = 0.05 for a 95% confidence interval, β = 0.2 for a power of 80%, and the non-inferiority limit (ΔL) corresponding to the greatest loss of effectiveness that is possible to consent [23]. The non-inferiority limit was conventionally set at − 10% based on previous studies [8, 13, 22]. We assumed that π*N =* 98% and πR = 88%. The sample size (*n* = 456) was increased by 10% to account for loss at follow-up, giving a final sample size of 530.

### Statistical analysis

MS Excel was used to construct a database to encode the data, and SPSS 20.0 (Chicago, IL) software was used for the analyses. First, descriptive statistics using the mean (standard deviation) or median (interquartile range) for a normal distribution or skewed distribution was computed, respectively. Next, the outcome measures in the two study arms were compared using Pearson’s chi-squared test for categorical data or Student’s *t-*test for means.

A one-sided Wald asymptotic test was used to assess the non-inferiority of the successful performance of the HIV self-test, correct interpretation of the HIV self-test result, requests for verbal assistance, retention rate, positive rate, linkage to care, and willingness to buy the HIV self-test kit if locally available in the UH arm, and these results were compared to those in the ADH arm. The confidence interval for the difference was based on the Wald asymptotic method, with an alpha level of 0.05 for 95% confidence limits. Non-inferiority was defined as a lower limit > − 10 of the 95% CI around the difference in outcomes. To determine the effects of interventions (UH versus DAH) on the primary and secondary outcomes, adjusted risk ratios (aRRs) calculated using Poisson regression were evaluated using two-sided statistical tests with a significance level set at *P <* 0.05. The participants who did not successfully complete the follow-up were not included in the analyses, as it was not possible to determine the primary and secondary outcomes for them.

Per cent agreement and Cohen’s κ coefficient were used to estimate the agreement between the participant-interpreted results and investigator-interpreted results. The degree of agreement was determined by Landis and Koch ranking (κ = 0: poor agreement; κ = 0.01–0.20: slight agreement; κ = 0.21–0.40: fair agreement; κ = 0.41–0.60: moderate agreement; κ = 0.61–0.80: substantial agreement; and k = 0.81–1.00: almost perfect agreement) [24].

Finally, the satisfaction was assessed using an arbitrary quantitative Likert scale containing four possible responses: 1 (most difficult), 2 (difficult), 3 (easy), and 4 (very easy) [25]. The mean and standard deviation for the Likert scale data were calculated and compared between the two arms using Student’s *t*-test.

### Ethics statement

This study received ethics approval from the ethics committee of the Health Public School of Kinshasa’s University. All participants provided written informed consent. No compensation was provided for participating in this study. The study was conducted by the Research, Teaching, and Care Unit of the Faculty of Medicine and Pharmacy of Kisangani’s University. This trial was retrospectively registered in the Pan African Clinical Trial Registry (www.pactr.org) database, ID number PACTR201904546865585.

## Results

### Recruitment and participant characteristics

Between August and November 2018, a total of 748 participants were assessed for eligibility. Of these, 530 were enrolled and randomized; however, follow-up was completed for 500 (94.3%): 263 (99.2%) in the DAH arm and 237 (89.4%) in the UH arm. Moreover, 28 participants were lost to follow-up in the UH arm, and 2 participants withdrew from study in the DAH arm (Fig. 1).

The characteristics of the participants in the two study groups were largely similar at baseline (Table 1). In brief, the participants were predominantly female, 25 to 49 years old, and currently single. The majority were students and had a university education level. All participants had evidence of high-risk behaviours, and more than four-fifths of the participants reported having unprotected intercourse with one or more partners in the past 6 months. Nearly half of the participants had been tested for HIV in the past, but the majority did not know about HIVST before this survey.

**Table 1:** Baseline characteristics of study participants by study arms

Participant characteristics	Directly assisted HIV self-testing (***N*** = 265)	Unassisted HIV self-testing (***N*** = 265)	Total (***N*** = 530)	***p***-value^***a***^
**Sex,** *n* (%)				0.859
Male	104 (39.2)	106 (40.0)	210 (39.6)	
Female	161 (60.8)	159 (60.0)	320 (60.4)	
**Age (years),** mean (SD)	26.9 (6.7)	26.8 (6.7)	26.9 (6.7)	0.881
**Age group,** *n* (%)				0.861
Younger 18 to 24 years	114 (43.0)	112 (42.3)	226 (42.6)	
Older 25 to 49 years	151 (57.0)	153 (57.7)	304 (57.4)	
**Marital status,** *n* (%)				0.589
Single	189 (71.3)	195 (73.6)	384 (72.5)	
Married/partnered	73 (27.5)	65 (24.5)	138 (26.0)	
Separated/divorced or widowed	3 (1.1)	5 (1.9)	8 (1.5)	
**Occupation,** *n* (%)				0.778
Student	141 (53.2)	136 (51.3)	277 (52.3)	
Employed	63 (23.8)	61 (23.0)	124 (23.4)	
Unemployed	61 (23.0)	68 (25.7)	129 (24.3)	
**Educational level,** *n* (%)				0.176
No formal education/ Primary school	37 (14.0)	53 (20.0)	90 (17.0)	
College or technical school	101 (38.1)	96 (36.2)	197 (37.2)	
University	127 (47.9)	116 (43.8)	243 (45.8)	
**Religion,** *n* (%)				0.355
Catholic Christianity	68 (25.7)	52 (19.6)	120 (22.6)	
Protestant or Pentecostal Christianity	77 (29.1)	80 (30.2)	157 (29.6)	
Islam	33 (12.5)	32 (12.1)	65 (12.3)	
Others	87 (32.8)	101 (38.1)	188 (35.5)	
**Recruited participants with the token distributing,** *n* (%)	88 (33.2)	98 (37.0)	186 (35.1)	0.363
**HIV transmission risk factor in the past six months**, *n* (%)				
Unprotected intercourse with one or more partners, or new sex partners	227 (85.7)	218 (82.3)	445 (84.0)	0.287
Commercial sex activity	73 (27.5)	86 (32.5)	159 (30.0)	0.218
Symptoms of sexually transmitted infections (STIs)	35 (13.2)	47 (17.7)	82 (15.5)	0.149
Being in a known HIV discordant partnership	6 (2.3)	3 (1.1)	9 (1.7)	0.313
**Previously tested for HIV,** *n* (%)				0.728
Never tested	135 (50.9)	131 (49.4)	266 (50.2)	
Ever tested	130 (49.1)	134 (50.6)	264 (49.8)	
**Previous knowledge about HIV self-testing,** *n* (%)				0.584
Yes	89 (33.6)	95 (35.8)	184 (34.7)	
No	176 (66.4)	170 (64.2)	346 (65.3)	

^*a*^Statistical comparisons were assessed by Pearson Chi-2 test or Student t test

### Practicability of the HIV self-test kit

As shown in Table 2, the rate of successfully performing the HIV self-test was high in both arms with no difference between them (93.2% in the UH arm versus 93.2% in the DAH arm). The rate of correctly interpreting of the HIV self-test result was 86.9% in the UH arm and 93.2% in the DAH arm, for an absolute difference of − 6.3% (95% CI: − 10.8 to 2.5); thus, non-inferiority was not demonstrated. The analysis of the interpretation of the self-test results in the two arms (Table 3) revealed that 25% of positive results were misinterpreted as negative in the DAH arm, whereas 16.7% of positive results were misinterpreted as invalid in the UH arm. Thus, overall, the Cohen’s κ coefficients for assessing the concordance between the results interpreted by the participants and the expected results were estimated at 0.69 and 0.44 for the DAH arm and the UH arm, respectively, indicating substantial agreement in the DAH arm and moderate agreement in the UH arm according to the Landis and Koch rankings.

**Table 2:** Characteristics of practicability and effectiveness of HIV self-testing in unassisted versus directly assisted approach and effects of unassisted approach on the practicability and effectiveness of HIV self-testing

Outcome	Directly assisted HIVST	Unassisted HIVST	Difference^***a***^ % (95% CI)	Non-inferiority of unassisted HIVST^***b***^	Adjusted Risk Ratio^***c***^ (95% CI)	***p-***value^***c***^
**Primary outcomes,** *n/N* (%)						
- Successful performance of HIV self-test	245/263 (93.2)	222/237 (93.7)	0.5 (−0.1 to 1.1)	Yes	1.11 [0.68 to 1.81]	0.817
- Correct interpretation of HIV self-test results	245/263 (93.2)	206/237 (86.9)	−6.3 (−10.8 to 2.5)	No	0.60 [0.36 to 0.98]	0.019
**Secondary outcome,** *n/N* (%)						
- Assistance requested	110/263 (41.8)	80/237 (33.8)^*d*^	−8.0 (−13.9 to 2.7)	No	0.87 [0.67 to 1.14]	0.063
- Retention rate^*e*^	263/265 (99.2)	237/265 (89.4)^*f*^	−9.8 (−15.9 to 4.9)	No	0.13 [0.03 to 0.51]	0.004
- Positivity rate with confirmation HIV testing	9/263 (3.4)	4/137 (1.7)	−1.7 (−6.1 to 2.7)	Yes	0.65 [0.32 to 2.02]	0.854
- Linkage to care	7/9 (77.8)	3/4 (75.0)	−2.8 (−9.5 to 5.1)	Yes	0.89 [0.42 to 1.89]	0.763
- Willing to buy HIV self-test kit if locally available^*g*^	195/263 (74.1)	217/237 (91.6)	17.5 (14.1 to 21.1)	Yes	4.20 [2.42 to 7.32]	< 0.001

^*a*^Difference assessed with Wald asymptotic test;

^*b*^Non-inferiority was defined as a lower limit > −10 of the 95% CI around the difference in outcomes;

^*c*^Estimates and confidence intervals are marginal effect from regression of Poisson;

^*d*^Majority (72/80; 90%) requested assistance via telephone; and 8 (10%) participants declared to have been assisted by a trusted person;

^*e*^Return rate was defined as the number of included participant who completed the evaluation throughout the follow-up period;

^*g*^The mean of self-test purchase price was estimated at 2.80 USD per test (limit: 0.33–5.41) in the unassisted HIV self-testing group while it was 2.96 USB per test (limit: 0.36–10.25) in the directly assisted group

*CI* Confidence internal, *HIVST* HIV self-testing

**Table 3:** Interpretation of self-test results in the hands of lay users compared to health care worker

		Directly assisted HIV self-testing				Unassisted HIV self-testing		
		Health care worker results				Health care worker results		
		Positive (*n* = 12)	Negative (*n* = 233)	Invalid (*n* = 18)		Positive (*n* = 6)	Negative (*n* = 216)	Invalid (*n* = 15)
Participant results	**Positive** (*n* = 12)	9 (75.0%)	3 (1.3%)	0 (0%)	**Positive** (*n* = 20)	5 (83.3%)	15 (6.9%)	0 (0%)
	**Negative** (*n* = 232)	3 (25.0%)	224 (96.1%)	5 (27.8%)	**Negative** (*n* = 195)	0 (0%)	190 (88.0%)	5 (33.3%)
	**Invalid** (*n* = 19)	0 (0%)	6 (2.6%)	13 (72.2%)	**Invalid** (*n* = 22)	1 (16.7%)	11 (5.1%)	10 (66.7%)
		**Estimate (% [95% CI])**				**Estimate (% [95% CI])**		
Agreement		93.5% [90.5 to 96.5]				84.4% [80.0 to 88.8]		
Cohen’s κ coefficient		0.69 [0.63 to 0.75]				0.44 (0.38 to 0.50)		

The effects of the follow-up intervention given after 72 h on the practicability of the HIV self-test were examined, and the participants in the UH arm had a significantly lower chance of correctly interpreting the test results than those in the DAH arm (aRR: 0.60 [95% CI: 0.36 to 0.98]; *P* = 0.019) (Table 2).

Finally, the participants’ satisfaction with the interpretation of the HIV self-test result was assessed using a Likert scale, and as shown in Table 4, the mean score was significantly higher in the DAH arm than in the UH arm (2.4/4 versus 2.3/4; *P* = 0.026).

**Table 4:** Results of the satisfaction questionnaire

Items	Directly assisted HIV self-testing (***N*** = 263)	Unassisted HIV self-testing (***N*** = 237)	Total (***N*** = 500)	***p-***value^**£**^
**Satisfaction questionnaire,** mean (SD)^*^				
- How did you find the identification of components of the kit	2.3 (0.7)	2.3 (0.7)	2.3 (0.7)	0.358
- How did you find the overall use of the HIV self-test	2.3 (0.5)	2.2 (0.5)	2.2 (0.5)	0.092
- How did you find the interpretation of HIV self-test result	2.4 (0.6)	2.3 (0.5)	2.3 (0.5)	0.026

^*^ The scale of response of satisfaction questionnaire was assessed by a Likert scale ranging from 1 (most difficult) to 4 (very easy); the results are mean ± 1 standard deviation (SD);

^£^ Statistical comparisons were assessed by Student *t* test for the comparisons of means

### Secondary outcomes

After the follow-up 72 h later for the participants in the UH arm who were performed the self-test kit at home or in a convenient private location, the rate of HIV positivity with the confirmatory test was 3.4% in the DAH arm and 1.7% in the UH arm. However, no significant difference was found between the two arms when evaluating these positivity rates as well as the requests for assistance and linkage to care (Table 2). However, the retention rate was significantly lower in the UH arm than in the DAH arm (89.4% versus 100%; difference: -10.6 [95% CI: − 18.9 to 2.9]; aRR: 0.13 [95% CI: 0.03 to 0.51]; *P* = 0.004). Willingness to buy an HIV self-test kit was higher in the UH arm than in the DAH arm (91.6% versus 74.1%; difference: 18.2 [15.1 to 21.8]; aRR: 4.20 [95% CI: 2.42 to 7.32]; *P* < 0.001) (Table 2). Finally, the mean purchase price of the HIV self-test was estimated at 2.80 USD in the UH arm and 2.96 USB in the DAH arm.

## Discussion

This study used a blood- and facility-based HIVST method to evaluate the practicability and effectiveness of HIVST between the UH and DAH approaches using a randomized, non-blinded, non-inferiority trial among a high-risk population for HIV infection acquisition in Kisangani, the DRC. The results of this study indicate that, in the cultural context of Kisangani, both UH and DAH had high rates of successfully performing the HIV self-test and correctly interpreting the HIVST results. Taken together, these findings indicate that the users of the UH approach had a significantly lower chance of correctly interpreting the test results than those who used the DAH approach. Additionally, our findings show that both UH and DAH can effectively scale up HIV testing and link seropositive individuals to care, even though the willingness to buy an HIV test was significantly higher in the UH arm.

In the DRC, the progress toward achieving the first 90 target has been slow. Thus, a major shift will be needed in the approach to testing to increase the effectiveness and efficiency of identifying those with an undiagnosed HIV infection [26]. Importantly, this study provides a better understanding of both approaches to delivering HIVST in terms of their practicability and effectiveness. The rate of successfully performing the HIV self-test was high in both arms in our study, and the error rate was not also different between the UH and DAH arms; this is contrary to what Asiimwe and colleagues found in Uganda, where a high error rate was observed when participants performed the oral test using the UH approach [18]. Furthermore, greater attention to training before testing may be needed to optimize the use of the HIV self-test kits using the DAH approach [9, 16, 18].

According to our findings, the agreement in the interpreted results was substantial between the self-testers and health-care workers in the DAH arm, with a Cohen’s k coefficient of 0.69, whereas it was moderate in the UH arm with a Cohen’s k coefficient of 0.44. Difficulties in interpreting the results have also been reported in the literature, with differences among the different approaches [8, 9, 13, 22]. The misinterpretation of positive results could negatively affect the control of the HIV epidemic [19] because HIVST is considered a test for triage [6, 26]. A recent systematic review reported that positive results were frequently misinterpreted as invalid (2.7 to 6.7%) in studies using the DAH approach, whereas in those using the UH approach, the reactive results were often misinterpreted as nonreactive (0.01 to 4.8%) [9]. Nevertheless, our findings indicate an opposite trend, with 25% of positive results misinterpreted as negative in the DAH arm and 16.7% of positive results misinterpreted as invalid in the UH arm. As previously demonstrated, a low educational level is the major factor associated with the misinterpretation of the self-test results aside from the testing approach (UH and DAH) [8, 11, 13].

The impact of HIVST in the continuum of care remains poorly understood in sub-Saharan Africa. A field study in Zambia demonstrated a high rate (90%) of linkage to care after HIVST. Although our study did not demonstrate the non-inferiority of the UH regarding the retention rate, Asiimwe and colleagues observed a difference between supervised HIVST and unsupervised HIVST regarding the retention rate in their study performed in Uganda. Indeed, they found that nearly 5% of the participants in the unsupervised HIVST group did not return to report their self-test results [18]. In our series, because of the confidential manner in which the self-tests were performed in the UH arm, the study team was not able to track the individuals lost to follow-up. Thus, further counselling may be needed to encourage individuals who self-test using the UH approach to return to the facility for confirmatory tests, post-counselling, prevention, and care. In this study, most participants in the UH arm who tested HIV seropositive were linked to care without a difference between the UH and DAH arms, thereby indicating the potential value of UH as a way to test and treat individuals living with HIV. Nevertheless, the monitoring and evaluation of UH present a real challenge in the DRC, where the health system remains very poor [27].

The cost of the HIV self-test kit has been identified as a potential barrier to adoption, willingness to use and purchase, and increasing HIVST, particularly among people in low-resource settings such as Congolese [28–30]. In this study, willingness to buy the HIV self-test kit was higher in the UH arm than in the DAH arm (91.6% versus 74.1%). This finding agrees with that from a systematic review by Figueroa and colleagues in which participants were more willing to pay for unsupervised HIVST than for supervised HIVST; the authors hypothesized that this finding was be due to the perception that DAH is similar to facility-based voluntary counselling and testing, which is often subsidized in public health care settings [9]. Furthermore, Mokgatle and Madiba showed that the willingness of students in South Africa to purchase a self-test kit was 74.7% without differentiating between UH and DAH [31]. Willingness to pay for the HIV self-test kit in this study may be over- or underestimated compared to actual HIV self-test kit purchasing behaviour. It is almost certain that the uptake of HIVST in the private sector in sub-Saharan Africa is far from optimal. Grants from governments, donors, and non-governmental organizations will be needed to maximize the uptake of HIV self-testing in the DRC, targeting key populations that must be served.

### Strengths and limitations

The strength of this study was the randomization procedure, which reduced the potential confounding factors between the study arms. To our knowledge, this is the first study in a French-speaking country in Africa to assess the practicability and effectiveness of UH versus DAH. A limitation of this study was that the re-reading by the research team of self-test device brought back by participants in sealed envelopes could have led to errors in interpreting the tests because other studies using the oral fluid-based self-test have shown that delayed re-reading of used oral self-tests is not currently a valid methodological approach to ensure quality and monitoring and may overestimate true HIV-positivity [32]. Our protocol involving re-reading the Exacto® HIV self-tests was validated in a preliminary investigation that assessed the stability of 30 performed self-tests (15 positives and 15 negatives), and no changes in the results were found 72 h after use.

## Conclusion

In conclusion, our study showed that UH is as practicable and effective as DAH among individuals at high risk for HIV infection in Kisangani, the DRC. Because errors in interpreting the self-test results and gaps in monitoring were found in the UH arm, additional support tools, such as instructional videos, the 24-h helpline, internet based-applications, and standard counselling prior to UH, need to be explored to improve the practicability and linkage to care. Taken together, UH, as well as DAH, should improve access to HIV testing in the DRC.

## Data Availability

All data generated or analysed during this study are included in this published article and its supplementary information files.

## Abbreviations

aRR: Adjusted risk ratio
CI: Confidence internal
DAH: Directly assisted HIV self-test
DRC: Democratic Republic of the Congo
HIV: Human immunodeficiency virus
HIVST: HIV self-testing
STI: Sexually transmitted infection
UH: Unassisted HIV self-test
WHO: World Health Organization
